# Development of the consensus-based recommendations for Podiatry care of Neuropathy In Cancer Survivors (PodNICS): a Delphi consensus study of Australian podiatrists

**DOI:** 10.1186/s13047-023-00632-0

**Published:** 2023-06-09

**Authors:** Sindhrani Dars, Elizabeth Buckley, Kerri Beckmann, David Roder, Helen Banwell

**Affiliations:** 1 Allied Health and Human Performance, University of South Australia, North Terrace, Adelaide, 5001 Australia; 2 Cancer Epidemiology and Population Health Research Group and Allied Health and Human Performance, University of South Australia, North Terrace, Adelaide, 5001 Australia

**Keywords:** Delphi survey, Consensus, Agreement, Podiatry, Chemotherapy Induced Peripheral Neuropathy

## Abstract

**Background:**

Chemotherapy Induced Peripheral Neuropathy (CIPN) is the most common presenting side effect of chemotherapy. As a sensory based neuropathy, this condition can persist for a long time after cessation of chemotherapy and impact the quality of life of cancer survivors. Podiatrists in Australia have been managing people with CIPN related lower limb complications, however guidelines on management of CIPN do not exist. The aim of this study was to achieve consensus and agreement of Australian podiatrists on strategies to best manage people presenting with symptoms of CIPN.

**Methods:**

An online three-round modified Delphi survey of Australian podiatrists with expertise in CIPN was conducted in line with recommendations for conducting and reporting of Delphi studies (CREDES). Panellists responded to open-ended questions in Round 1, whereupon their responses were themed into statements and analysed for existing consensus. Statements not reaching consensus were returned during Round 2 to seek agreement from responders using a five-point Likert scale and to allow responders to make further comments. For a statement to reach consensus or agreement, 70% or more of panellists needed to make the same comment or agree or strongly agree with the same themed statement. Statements reaching 50 to 69% consensus or agreement were returned to panellists in Round 3 for them to consider their responses in the light of group outcomes.

**Results:**

Round one resulted in 229 comments from 21 of 26 podiatrists who agreed to participate. These comments were themed into 53 statements with 11 consensus statements accepted. Round 2 resulted in 22 statements reaching agreement, and 15 new statements being generated from 18 comments made by 17 respondents. Round 3 resulted in 11 statements reaching agreement. Outcomes were developed into a set of clinical recommendations for diagnosis and management of people presenting with CIPN. These recommendations provide guidance on 1) identifying common signs and symptoms of CIPN including sensory, motor and autonomic symptoms; 2) diagnosis and assessment of CIPN including neurological, motor and dermatological assessment modalities; and 3) best clinical practice and management strategies for CIPN identified by podiatrists including both podiatry and non-podiatry specific care.

**Conclusions:**

This is the first study in podiatry literature to develop expert-informed consensus-based recommendations for clinical presentation, diagnosis and assessment and management of people with CIPN. These recommendations aim to help guide podiatrists in the consistent care of people with CIPN.

**Supplementary Information:**

The online version contains supplementary material available at 10.1186/s13047-023-00632-0.

## Background

Chemotherapy Induced Peripheral Neuropathy (CIPN) is a chronic, debilitating consequence of chemotherapy affecting approximately 60% of people undergoing cancer treatment [1–3]. It is commonly caused by neurotoxic chemotherapy, mainly platinum and taxane based compounds, which are widely used for breast and colorectal cancer [4–6]. The development of CIPN has been identified as a leading cause of chemotherapy dose modification or reduction in 17–85% of people receiving chemotherapy [7, 8] and complete cessation in 4–16% of cases [9, 10]. CIPN, along with the associated neuropathic pain, can persist in 31–49% of cancer survivors for up to three years post chemotherapy, due to a phenomenon known as “coasting”, which is a worsening of symptoms after cessation of the chemotherapy regime [11–13]. CIPN can present as a mixed neuropathy with various effects on the body including motor, autonomic and sensory disturbances [14], however, the sensory nerves are reported to be most affected [14–16].

Sensory neuropathy is frequently associated with deterioration in lower limb health and overall quality of life for cancer survivors [17]. Specifically, CIPN can present as a loss of protective sensation in feet leading to ulcerations and in the some cases, amputation [18]; reduced proprioception leading to ataxic gait and increased risk of falls [19, 20]; neuralgia (nerve pain including numbness, allodynia and tingling) presenting in a “glove and stocking” pattern [15, 21]; changes in skin integrity leading to dermatological problems such as blistering, skin crusting and peeling, hyperkeratosis and changes in sweat production [22]; and changes in nails presenting as pathologies like onychocryptosis (ingrown toenail), paronychia (infection around nail), onycholysis (separation of nail from nail bed) and cessation of nail growth with ‘unsightly’ dystrophic changes [22–24].

A diagnosis of CIPN can be made using a variety of valid and reliable tools, including: the National Cancer Institute Common Terminology Criteria for Adverse Events (NCI-CTCAE); the European Organization for Research and Treatment of Cancer as Chemotherapy-Induced Peripheral Neuropathy 20 (EORTC CIPN 20); and the Quality-of-Life Questionnaire (EORTC QLQ-C30); the Total Neuropathy Score (TNS) and its different versions; and Functional Assessment of Cancer Therapy (FACT) and its versions. Podiatrists frequently assess for peripheral neuropathy using neurophysiological examinations, such as using a 10-gram Semmes Weinstein monofilament to assess protective sensation, and Neurothesiometer or a 128C Hz tuning fork to assess vibration sensation, as a proxy for proprioceptive sensation [13, 25–27]. What is not clearly understood, however, is if podiatrists also use these methods to assess for CIPN, use alternative methods, or rely on pre-existing diagnoses from referring practitioners.

Management of the symptoms associated with CIPN has also proven challenging. A review of current literature indicates that no known treatment options have been identified as effective for CIPN, nor are there any protocols or agents that can successfully prevent CIPN [13, 28]. Currently there is very little understanding of how patients with CIPN-induced lower limb problems are managed in the podiatry setting and little evidence available to guide practitioners on the management of CIPN in cancer survivors. Not unsurprisingly, most of the research on lower limb neuropathy is focused on the more common consequences of diabetes-related foot disease, with evidence-based guidelines in place to direct management by podiatrists [29, 30].

Given that the 5-year relative survival rate for all cancers in Australia between 2014–2018 was 70% and rising [31], there will be increasing numbers of cancer survivors living with the consequences of chemotherapy. It would be prudent for our profession to have recommendations in place to guide practice associated with CIPN to help improve the care of these patients.

Anecdotally, podiatrists report that cancer survivors with CIPN-induced lower limb changes appear to seek their services for assistance with foot health. It is assumed these patients are most frequently managed within private or community-based podiatry clinics due to the scarcity of specialised clinics providing transition and follow up care for patients with cancer and a focus on diabetes-related foot disease within ‘high-risk’ foot clinics [18]. However, little is known on where they attend, their rate of attendance, referral sources and funding mechanisms that support these patients. Understanding these factors can assist in service planning, particularly in consideration of increasing cancer survivorship numbers.

In the absence of clinical evidence, this study aimed to explore the experiences of Australian podiatrists and garner consensus and agreement from those with experience and expertise in CIPN, and develop a set of clinical recommendations that may guide contemporary clinical practice in light of informed care and current knowledge.

## Methods

### Study design

A modified three round Delphi survey was undertaken in line with recommendations for conducting and reporting Delphi studies – CREDES [32]. Delphi methodology involves a panel of experts being anonymously and individually surveyed over a series of iterations (known as Rounds) to gather consensus and agreement on topics that are least explored and lack theoretical frameworks [33, 34]. It has been widely used in different healthcare settings to formulate consensus-based recommendations and guidelines for clinical implementation [35–37]. This study method also has the benefit of being able to be completed in its entirety online, removing the requirement for people to be geographically available. For this survey, all three rounds were conducted via the online platform Survey Monkey® [38] with the survey links distributed to panellists via email. This study was approved by the University of South Australia’s Human Research Ethics Committee (Protocol number 204307).

### Panellists

Potential panellists in addition to being a registered podiatrist, satisfied at least one of the following criteria to be eligible for the study:

- a) has worked within a high-risk foot clinic for 5 years or more, or chronic disease focused private setting for 10 years or more, or
- b) holds an academic position teaching podiatry led neuropathy-based management techniques, or
- c) has published on conservative intervention techniques for lower limb neuropathy within the last 5 years

No incentives were offered, and panellists were aware they could withdraw their consent to be involved at any stage. However, to improve the robustness of the outcomes, panellists were asked to commit, and respond independently, for each round.

### Survey development

Round 1 of the survey was purpose-built by members of the authorship group who are clinical and academic podiatrists (SD, HB), with input from authors with experience in cancer-related research (LB, DR). The survey was piloted by three independent external reviewers (two podiatrists and one physiotherapist), with wording and functionality modified based on their feedback. The podiatrists who piloted the questionnaire did not participate further in the study.

In Round 1 (Additional file 1), panellists were asked to complete a short section on their professional experiences (clinical, research and academic) to ensure they met the eligibility criteria. They then provided responses to questions regarding their demographics, opinions and practice habits in relation to CIPN. Demographic questions included gender, age and information regarding their podiatry qualifications. Questions about their experience included years of practice, practice location, average practice hours in primary and secondary positions. In the subsequent sections of the survey, panellists were asked questions regarding:

1. Clinical factors and presentation of people with CIPN
2. Diagnosis and assessment of CIPN
3. Podiatry management of CIPN

For this section of Round 1, questions were purposely open-ended to encourage opinion sharing, with one exception where panellists used a five-point Likert scale i.e., strongly disagree, disagree, neutral, agree or strongly agree to identify their confidence with various assessment tools to diagnose CIPN.

Round 2 statements were developed based on the outcomes of Round 1 (as described below). Round 2 also offered panellists the opportunity to add additional opinions and practice habits in relation to the management of clients with CIPN.

Round 3 statements were developed based on the responses to the previous two rounds. No further comments were allowed in this round.

### Procedure

Podiatrists were alerted to the study via passive and active contact methods; passively via personal social media accounts of the authors and national advertising via the Australian Podiatry Association, and actively via targeted emails and invitations sent to the heads of podiatry departments of public health settings and educational institutes for dissemination amongst staff. To minimise location and experience bias, this study aimed to recruit a panel of 25 podiatrists from a variety of settings and locations, specifically aiming for 10 from the public sector, 10 from private and 5 from an academic or research background. Similar sample sizes have been considered adequate and feasible for Delphi surveys and are consistent with other podiatry specific Delphi studies [35, 39, 40]. Podiatrist Registrant Data from 2021 [41] was used to ensure recruitment was geographically balanced to include five panellists from Victoria and New South Wales, three from South Australia, Western Australia, and Queensland, and two from Tasmania, Australian Capital Territory and Northern Territory.

Both panellist eligibility and written informed consent were confirmed at the start of the online survey for Round 1, with skip logic employed to exclude those who did not consent or meet the criteria. Panellists were given four weeks to complete each survey round with reminder emails sent one week prior to the closing date. At the end of each round, all data was downloaded from SurveyMonkey™ into Microsoft Excel (Microsoft Corp, Redmond Washington) for analysis. All three rounds of the Delphi were conducted between April and September 2022.

### Analysis

Open-ended responses to Round 1 were subjected to inductive quantitative content analysis allowing comments made by panellists to be considered individually and aligned into statements [42, 43]. During this review process comments were either aligned with an existing theme or generated into a new theme, with previously reviewed comments able to be re-coded as themes emerged. To minimise bias, analysis was independently conducted by two authors (SD and HB) with any conflicts resolved by discussion. All emergent themes were then converted into a series of statements for circulation in further rounds.

The a-priori decision was to set the level of consensus and agreement at 70%. Therefore, for a statement to be accepted as a consensus, ≥ 70% of panellists were required to make the same ‘themed statement’ in response to an open-ended question (available in Round 1 and 2 only). For a statement to be accepted as reaching agreement, ≥ 70% of panellists were required to indicate they agreed or strongly agreed to that statement on a five-point Likert scale. Statements that reached between 50 and 69% consensus or agreement were retained for further consideration in the subsequent rounds (where available) to allow responders to reconsider them in light of group responses. Statements with less than 50% agreement in Round 1 and 2 and less than 70% agreement in Round 3 were excluded. This method of statement theming and the levels of consensus and agreement are consistent with similar Delphi studies [39, 40, 44].

Outcomes of each round were reviewed by the full authorship team. Collated outcomes from group responses in previous rounds were presented to the panellists within the subsequent round, where available. The number of panellists that had identified or agreed with statements in each round are provided in the supplement (Additional file 2).

### Development of consensus-based recommendations for Podiatry care of Neuropathy In Cancer Survivors (PodNICS)

The final accepted statements were organised to align with the SOAP (Subjective, Objective, Assessment and Plan) format which is not only useful in documenting clinical findings but also serves as a cognitive framework for clinical reasoning to assess, diagnose and treat a patient in health care settings [45]. Podiatry care of Neuropathy In Cancer Survivors (PodNICS) was developed by the clinical podiatrists within the research team (SD and HB) from the final accepted statements as clinical recommendations to guide the care of clients with CIPN seeking podiatry services.

Usability has been identified as a desirable factor of clinical decision support systems that include guidance on diagnosis and treatment of chronic conditions [46]. To this end, the PodNICS was reviewed by four independent podiatrists for its structure, functionality and to ensure it well-incorporated all accepted statements from the three rounds.

All authors also checked the final recommendations for ease of use and implications in the clinical practice.

## Results

### Panellists’ characteristics

Twenty-six out of 76 invited podiatrists consented to participate, met the eligibility criteria, and were enrolled into the study. Twenty-one of those recruited completed Round 1, (four males and 17 females with the mean age of 43.8 years). The geographical representation of the panellists did not meet the planned distribution; however, each State and Territory was represented by at least one participating podiatrist. The mean podiatry experience among panellists was 19 (± 8) years, and all had worked within a high-risk foot clinic for five years or more or chronic disease focused private setting for 10 years or more. Table 1 outlines panellist’s characteristics in detail.

**Table 1:** Panellist characteristics

Category	Total number or mean	Percentage or standard deviation
Gender	4 Males	19%
	17 Females	81%
Age	44 years	+ 8 years
Practice duration	19 years	+ 8 years
Highest qualification	3 PhD	14%
	1 Professional doctorate	5%
	2 Master’s degree	9%
	7 Graduate Diploma	33%
	5 Graduate Certificate	24%
	3 Bachelor's degree	14%
Primary Position	14 Clinicians	67%
	2 Researchers	9%
	5 Manager post in Academic or Private clinical settings	24%
Primary Practice location	1 Australian Capital Territory	5%
	1 New South Wales	5%
	1 Northern Territory	5%
	5 Queensland	24%
	7 South Australia	33%
	1 Tasmania	5%
	4 Victoria	19%
	1 Western Australia	5%
Secondary Position	5 Clinicians	24%
	2 Academics	9%
	2 Project Managers	9%
	1 Director of private practice	5%
	1 Consumer advisor for clinical trials	5%
Estimated weekly workload in primary position	35 h	+ 9 h
Estimated weekly patient load	27 clients	+ 18 clients

### Panellists experience with clients presenting with CIPN

Fifteen of the 21 panellists (71%) reported they had seen clients with CIPN in the last year, with around 33% of them seeing more than five clients in the three months prior to completing the survey. The most common referral source for CIPN clients was their general practitioner (65%), with other referral sources including self-referral (35%), oncologists (29%), alternative allied health or exercise professionals e.g., physiotherapist, exercise physiologist, speech pathologist etc. (29%), and nurse practitioners (18%). Referrals from My Aged Care, hospital outpatients and neurologists were also identified once each (6% respectively) (Table 2).

**Table 2:** Panellists experience with clients with CIPN

Category	Total number	Percentage
Seen clients with CIPN in the last year (*n* = 21)	15 Yes	71%
	5 No	24%
	1 No response	5%
Estimated average number of clients with CIPN seen in the last 3 months (*n* = 15)	10 (0–5 clients with CIPN)	67%
	2 (5–10 clients with CIPN)	13%
	2 (10–15 clients with CIPN)	13%
	1 (15–20 clients with CIPN)	8%
Referral sources for clients with CIPN (*n* = 17)	11 General Practitioner	65%
	6 Self-referred	35%
	5 Oncologist	29%
	5 Allied health or exercise professionals e.g., Physiotherapist, Exercise Physiologist, Speech pathologist etc	29%
	3 Nurse Practitioners	18%
	1 My Aged Care	6%
	1 Hospital Outpatients	6%
	1 Neurologist	6%
Common funding sources to attend Podiatry services for people with CIPN (*n* = 20)	12 Publicly funded (e.g., attending a hospital or community-based practice)	60%
	5 Chronic disease management plan	25%
	CDMP/Medicare	10%
	2 Private health	5%
	1 Commonwealth home support program and home care package	
Reason for management choices (*n* = 17)	8 Clinical experience and previous success to management options	47%
	4 Learning from senior/ experienced podiatry colleagues	23%
	4 Knowledge crossover from managing Diabetes related foot concerns	23%
	2 Learning from other professionals including neurologists, diabetes specialists, oncology nurses and pain specialists	12%
	2 Patient feedback	12%
	2 Current evidence	12%
	2 Anecdote and learnt skills (unknown source)	12%
	1 Learnt skills from entry-level podiatrists who retain knowledge of modern interventions learnt fresh from university	6%

With respect to common funding sources for the provision of podiatry services, 60% of responders identified their clients as being publicly funded (e.g., attending a hospital or community-based practice). Chronic disease management plan (CDMP) Medicare funding was the second most common source (25%) followed by private health funding (10%) and Commonwealth home support program and home care package (5%) (Table 2).

There were mixed reports of how often clients presented with an existing CIPN diagnosis versus a diagnosis made by the responder. Eight podiatrists indicated that more than 80% of their clients with CIPN presented with an existing diagnosis made by other professionals like neurologists and GPs. However, five respondents reported less than 10% of clients having an existing diagnosis of CIPN. Half of the panellists indicated that they had diagnosed one or more clients with CIPN within the last year who were unaware of their diagnosis.

When asked about their choice of management rationale (in the absence of guidelines) for podiatry management of CIPN, eight common themes were identified from 25 statements by 17 podiatrists (Table 2). The most common rationale identified was clinical experience and previous success from management (47%) followed by knowledge crossover from managing diabetes related foot concerns (23%) and learnt skills from other experienced podiatrists (23%).

### Survey findings

**Fig. 1:**
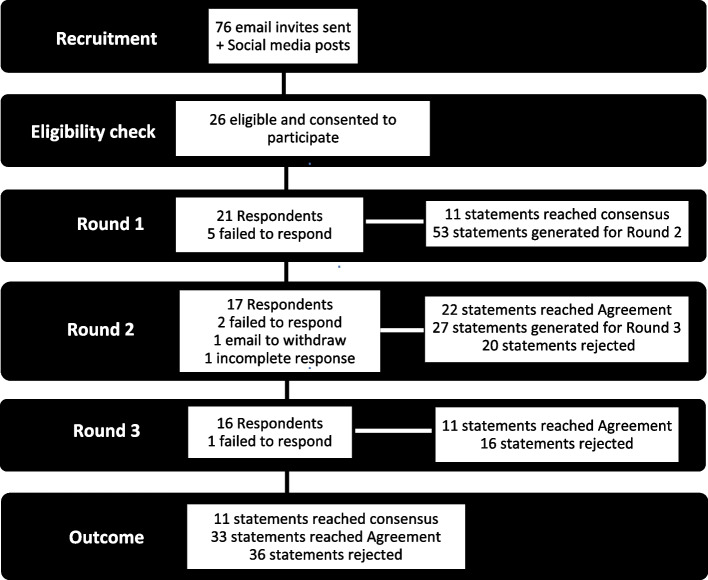
Flow diagram of the process of Delphi with the outcome

Figure 1 outlines the process of Delphi rounds and the respective outcomes.

### Round 1

Round 1 resulted in 229 comments on the open-ended questions that were themed into 53 statements. Of these, 11 statements reached consensus (Table 3) and 42 statements were returned to panellists for consideration on agreement in Round 2 (Additional file 2). No statements were excluded in Round 1.

**Table 3:** Statements reaching consensus or agreement within three rounds

Category	Statement	Round accepted	(*n* = X/X no. of panellists)—% consensus/agreement
**Clinical factors and presentation of people with CIPN**			
**Common presenting signs and symptoms of people with CIPN**	Sensory symptoms such as neuralgia, dysesthesia (abnormal sensation), paraesthesia (pins and needles), allodynia (abnormal response to stimulus) and/or hyperesthesia (exaggerated pain response)	One	(*n* = 18/19) – 95% consensus
	Loss of protective sensation (LOPS) and Loss of proprioception	Two	(*n* = 13/17) – 76.5% agreement
	Autonomic changes including but not limited to: blood pressure and temperature regulation (cold feet/Raynaud's phenomenon)	Two	(*n* = 13/17) – 76.5% agreement
	Nail changes including but not limited to: onychogryphosis, onychomycosis, Onychauxis, Onychocryptosis and nails that are friable, dystrophic, have reduced growth and flaking	Two	(*n* = 13/17) – 76.5% agreement
	Skin changes including but not limited to; atrophy + rubor, skin shedding/peeling, dry skin, moccasin type cracking and painful blistering	Two	(*n* = 13/17) – 76.5% agreement
**Clinical factors or presentation unique to CIPN**	Sudden (acute) onset and quick progression of symptoms	Two	(*n* = 14/17) – 82% agreement
	In some people, symptoms may improve or resolve with chemotherapy dose reduction or cessation	Two	(*n* = 13/17) – 76.5% agreement
	Skin anhidrosis with rubor, skin shedding and increased injuries	Three	(*n* = 12/16) – 75% agreement
**Additional information on Clinical factors and presentation of people with CIPN**	Can reduce patient's confidence and engagement in physical activity	Three	(*n* = 12/16) – 75% agreement
**Diagnosis and Assessment of CIPN**			
**Diagnostic and Assessment tools routinely utilised**	Monofilament (10 g)	One	(*n* = 16/18) – 89% consensus
	Tuning fork (128 Hz) or graduated	One	(*n* = 14/18) – 78% consensus
	Medical history and Subjective questioning including client reported signs and symptoms (changes to sensation), Visual Analogue Scale (VAS), and Quality of Life (QOL) questionnaires	One	(*n* = 13/18) – 72% consensus
	Inspect for Callus, pre-ulcerative lesions and ulcers	Two	(*n* = 15/17) – 88% agreement
	Changes to skin integrity following chemotherapy	Two	(*n* = 13/16) – 81% agreement
	Muscle strength and Joint Range of Motion	Two	(*n* = 12/17) – 70.6% agreement
	Diabetes foot assessment	Three	(*n* = 14/16) – 87.5% agreement
	Footwear assessment	Three	(*n* = 13/16) – 81% agreement
	Deep Tendon reflexes	Three	(*n* = 13/16) – 81% agreement
**Assessment tools/pathways that could confirm diagnosis**	Oncologist notification	One	(*n* = 17/18) – 94% consensus
	10gm Monofilament test	One	(*n* = 16/18) – 89% consensus
	Presence of wounds/ulcers due to unfelt trauma	One	(*n* = 16/18) – 89% consensus
	Self-reported neurological symptoms	One	(*n* = 15/18) – 83% consensus
	Presence of comorbidities likely to worsen neuropathy e.g., diabetes	One	(*n* = 15/18) – 83% consensus
	Tuning fork assessment	One	(*n* = 14/18) – 78% consensus
	GP notification	Two	(*n* = 15/17) – 88% agreement
	Nerve conduction study	Two	(*n* = 14/17) – 82% agreement
	Patient reported diagnosis	Two	(*n* = 13/17) – 76.5% agreement
	Biothesiometer or Neurothesiometer	Two	(*n* = 13/17) – 76.5% agreement
	Patient reported signs and symptoms/outcomes using validated questionnaires e.g., Visual Analogue Scale (VAS)	Two	(*n* = 13/17) – 76.5% agreement
**Podiatry Management of CIPN**			
**Podiatry Management of CIPN**	Education including, changes to sensation, skin and nails, and how to avoid complications e.g. regular self-check of feet, avoid bare feet, regular emollient, use of socks and shoes. Education also on importance of regular neurological screens by professionals like podiatrist or neurologist	One	(*n* = 14/18) – 78% consensus
	Management and offloading of pressure lesions, wounds or blisters	Two	(*n* = 16/16) – 100% agreement
	Communication with GP and oncology team, particularly where foot-related symptoms are severe	Two	(*n* = 16/16) – 100% agreement
	Advise on escalation of care if needed in case of development of foot infection or ulceration	Two	(*n* = 16/16) – 100% agreement
	Footwear assessment and education (properly fitting, supportive, light weight and comfortable)	Two	(*n* = 15/16) – 94% agreement
	Engagement with possible referral to other allied health professionals as required (e.g., Physiotherapist, Occupational therapist, Exercise physiologist, psychologist and pain management clinics)	Two	(*n* = 15/16) – 94% agreement
	Assessing that pharmacological pain management is in place and educate on non-pharmacological pain management modalities (heat packs, wheat bags, topical capsaicin etc.)	Two	(*n* = 13/16) – 81% agreement
	Regular footcare (nails including ingrowing toenails and skin including hyperkeratosis)	Two	(*n* = 13/16) – 81% agreement
	A targeted personalised management plan appropriate for severity of the condition and considering patient's finances	Two	(*n* = 13/16) – 81% agreement
	Discuss options for use of mechanical aids like walkers and braces	Three	(*n* = 14/16) – 87.5% agreement
	Discussion regarding their driving ability	Three	(*n* = 13/16) – 81% agreement
	Advising on appropriate physical activity or exercise regimes	Three	(*n* = 13/16) – 81% agreement
	Advise on lifestyle changes including alcohol, smoking, and diet	Three	(*n* = 12/16) – 75% agreement
**Additional information on Podiatry management of CIPN**	Multidisciplinary care is essential	Three	(*n* = 14/16) – 87.5% agreement
	Podiatrist-based resources on the management of CIPN are required	Three	(*n* = 12/16) – 75% agreement

Statements accepted for consensus included one in relation to Clinical factors and presentation of people with CIPN, nine statements in relation to Diagnosis and Assessment of CIPN, and one statement regarding Podiatry Management of CIPN (Table 3).

### Round 2 and 3

Round 2 was completed by 17 panellists, with one panellist withdrawing via email, two failing to respond and one incomplete response received. Round 2 resulted in 22 statements reaching agreement; 12 statements receiving between 50 and 69% agreement requiring further review in Round 3 (Additional file 2); and 20 statements being excluded for not reaching 50% or more agreement (Additional file 2). A further 18 new comments were received that were themed into 15 new statements for Round 3 (Additional file 2).

Of the statements reaching agreement, six related to the clinical factors and presentation of people with CIPN, eight statements were in regarding to diagnosis and assessment of CIPN, and eight statements related to podiatry management of CIPN.

One further panellist failed to complete Round 3 (*n* = 16). Round 3 resulted in 11 statements reaching agreement and 16 statements being excluded for not reaching 70% or more agreement (Additional file 2). Of the statements reaching agreement, two related to the clinical factors and presentation of people with CIPN, three statements related to the diagnosis and assessment of CIPN, and six statements related to podiatry management of CIPN.

### Key findings from all three rounds

Table 3 outlines all the statements reaching consensus and agreement from all three rounds of the Delphi. With respect to the presentation of CIPN, the Delphi panel identified the presence of sensory symptoms such as neuralgia, paresthesias and allodynia (95% consensus). Other common presenting symptoms identified by the panel were autonomic changes such as changes in temperature regulation leading to cold feet/Raynaud’s phenomenon. Disruption of physical activity and engagement caused by reduced patients’ confidence was also agreed as one of the presentations for people with CIPN.

The objective assessment tools to diagnose CIPN agreed by the panel included monofilament for LOPS (89% consensus); tuning fork (78% consensus), biothesiometer (76% agreement) and nerve conduction study if required for loss of proprioception (82% agreement); deep tendon reflexes (81% agreement), joint range of motion (ROM) and muscle strength assessment (71% agreement).

The panel agreed on the importance of tailoring management according to the severity of CIPN and identified various aspects of patient care. Agreement on management practices not only included management of problems specific to the lower limb such as skin lesions, pain and footwear but also holistic care of patients through discussions about lifestyle, physical activity and overall pain management. For the management of foot specific problems, the panel agreed on the need to assess the skin integrity for any pre-ulcerative (hyperkeratosis and helomas) and ulcerative lesions (88% agreement) and subsequent podiatry management by offloading and footwear modifications as required (100% agreement). The panel also indicated the importance of patient education including: changes in sensation, skin and nails and their implications for foot health; basic foot care e.g. regular self-check of feet, avoiding bare feet, regular emollient, use of socks and shoes (78% consensus); and noted the need for escalation of care advice when required such as in an instance of infection or ulceration (100% agreement). Regular neurological screens by professionals as podiatrists or neurologists were also advised (78% consensus). Panellists also encouraged discussion with the clients around their overall health and lifestyle with possible recommendations on diet, smoking and alcohol use; ability to participate in Activities of Daily Living (ADLs); overall mobility with possible discussion on use of mobility aids; and their ability to drive a motor vehicle with presence of neuropathy (75–81% agreement). They also recommended that podiatry-specific resources on the management of CIPN be accessible to the clients as a part of their care (75% agreement).

The involvement of a multidisciplinary team in the care of the patient was strongly supported, with ongoing communication with the patient's GP, oncologist and other health care professionals involved in their care (100% agreement). Referrals to other allied health professionals such as physiotherapists, occupational therapists, pain specialists and psychologists as per patient needs were also encouraged (94% agreement).

### Consensus-based recommendations for Podiatrists informed care of Neuropathy In Cancer Survivorship (PodNICS)

The Delphi process produced a total of 44 accepted statements which informed the development of the Podiatry care of Neuropathy In Cancer Survivors (PodNICS) as clinical recommendations to guide the care of clients with CIPN seeking podiatry services. Recommendations are organised into four categories: Signs and symptoms of CIPN; Diagnosis and assessment of CIPN; Management strategies of CIPN, and Further considerations.

**Fig. 2:**
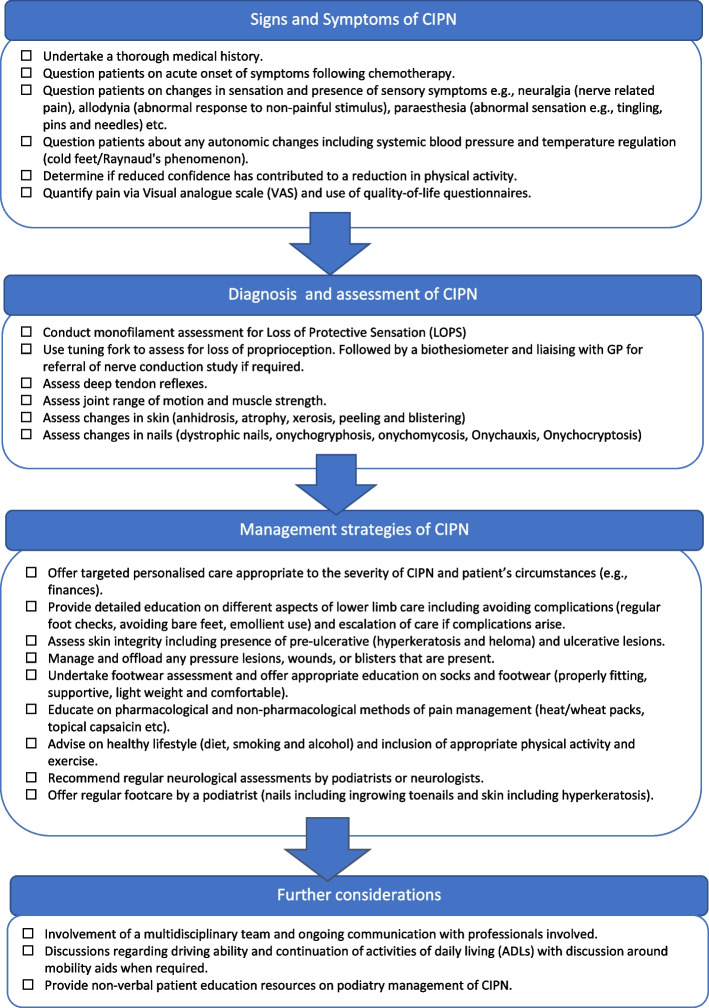
Podiatry care of Neuropathy In Cancer Survivors (PodNICS)

The review of the draft PodNICS by independent podiatrists confirmed that it incorporated results of the Delphi surveys faithfully and was relevant to clinical practice, with only minor changes recommended for wording of the draft. The final PodNICS incorporating the suggested changes of the external reviewers is shown in Fig. 2.

## Discussion

This Delphi study aimed to gather the expertise of experienced Australian podiatrists to identify contemporary and informed practice in assessing and managing CIPN within the podiatry setting. To the best of our knowledge, this is the first study to gather and collate expert opinion to inform clinical recommendations for the podiatry management of clients with CIPN. As such, it is an initial step toward establishing consistency in clinical practice and directing future investigations on efficacy of podiatry-led management strategies specific for this form of neuropathy. Encouragingly, this process highlighted that many podiatrists are already working with evidence-based recommendations for identifying neuropathy, and there were consistencies noted in relation to current practice.

Fifty percent of the panel reported making at least one or more new diagnoses of CIPN in their clinical practice in people with chemotherapy exposure but unaware of their neuropathy. This is a critical finding suggesting that podiatrists could be the first health practitioners to identify their neuropathy and manage it accordingly. There was consensus amongst the panel on objectively diagnosing CIPN using monofilament for LOPS; tuning fork, biothesiometer/Neurothesiometer and nerve conduction study if required for loss of proprioception; deep tendon reflexes and muscle ROM and strength. These assessments are established evidence-based, valid and reliable methods for diagnosing diabetes related peripheral neuropathy [30, 47, 48]. As identified by panellists, in absence of current guidelines on podiatry management of CIPN, their choices for CIPN management often rely on the knowledge crossover from managing diabetes related foot concerns. It should also be considered that despite the causes being different, the resulting problems are similar between CIPN and diabetes and hence the assessment methods can be similar. Our panel also recommended assessing ROM and strength which are both biomechanical contributors to the development of foot deformities leading to increased risk of pre-ulcerative lesions (hyperkeratosis) and consequent ulcerations [30].

The panel was also quite consistent in identifying the commonly presenting symptoms of CIPN including changes to sensation, skin and nails; presence of LOPS; and lack of proprioception. This presentation does not differ from diabetes related neuropathy but has been identified to be related to CIPN in the literature [14, 21–24]. However, a presentation unique to CIPN identified by our panel, that is not common with other types of neuropathies, is sudden/acute onset of symptoms. This could be explained by the development of CIPN being highly correlated with exposure to dose intense chemotherapy for cancer treatment [49, 50]. It was also agreed by panellists that in some people the symptoms may subside with chemotherapy dose reduction or cessation. This again is supported by current literature showing dose reductions or complete chemotherapy cessation can alleviate acute neuropathy symptoms [7, 8]. Within the Delphi panel, there was agreement on skin xerosis, shedding and rubor with increased injuries being unique to CIPN clients. Several authors in the past have also identified these skin changes as side effects of CIPN [22–24]. One of the manifestations of CIPN identified in the literature is increased risks of falls [20, 51] though this was not directly identified in our Delphi study. However, loss of balance and proprioception due to CIPN were identified, which eventually can lead to increased falls risk.

Most of the research and practice guidelines on CIPN management focus on pharmacological prevention and/or treatment with no definitive evidence on effectiveness of any neuroprotectors or treatment modalities [55–57]. There is limited research on the non-pharmacological CIPN management involving allied health care professionals and unfortunately, none of the research or current guidelines include podiatry care as a part of CIPN management [13, 19, 28, 58]. There are several CIPN related lower limbs consequences that can impact the quality of life of cancer survivors such as increased falls risks due to reduced proprioception, affected balance, gait, mobility and functionality, and increased risk of development of ulcerations due to changes in sensation [17, 19, 20, 51]. These consequences presenting with other conditions such as diabetes have been effectively managed by podiatrists. Podiatry intervention has been reported to significantly reduce the occurrence of lower limb amputations caused by diabetes [59]. The similar preventative interventions are highly likely to be effective to care for people with CIPN. Moreover, in the general population, core podiatry including treatment of nails, hyperkeratosis and helomas, footwear intervention and foot health advice by podiatrists have previously been proven effective in alleviating pain, reducing falls risk and greater patient satisfaction [60–64]. The common podiatry management shown to reduce the risks of falls include footwear modification, foot and ankle exercises and education [64, 65]. The management strategies for CIPN identified by the panel included footwear education and advice on physical activity.

Alternate and adjunct treatments suggested by a few podiatrists in the panel which didn’t reach agreement included cryotherapy, scrambler therapy, acupuncture and massage therapy. Given the lack of or low-quality evidence for their effectiveness [13, 19, 28, 55] such therapies are not currently recommended as part of the standard care. However, further research into their potential is needed so that more comprehensive guidelines for effective treatment can be developed in future to ensure optimal care of people with CIPN by the podiatrists.

Development of clinical pathways in different health settings have been shown to reduce fragmentation and variation in clinical practice and improve patient outcomes [66]. A meta-analysis of studies on the use of clinical decision support systems for the preventative care services has demonstrated their effectiveness [67]. The clinical recommendations developed from this Delphi includes a logical order of events that should occur in a clinical setting to manage a client presenting with any new/reoccurring complaint due to CINP. This logical order covers all important aspects of clinical reasoning by incorporating the subjective history, objective assessments and subsequent care plan for the patient based on the gathered information.

### Limitations and future research directions

The Delphi survey methodology, even though a well-known and widely researched methodology, is sometimes criticised due to the quantitative nature of the data being collected and its purposeful sampling methodology [32, 34, 68]. However, with panellists recruited from different geographical locations with the collective clinical experience of 19 years, a broad range of knowledgeable opinions were collected to provide evidence of consistency in podiatry practice. The definition of an ‘expert podiatrist’ is consistent with previous Delphi studies conducted in the field of podiatry [35, 39, 40, 44] and respondents remained anonymous to each other throughout the survey. However, the management options indicated in the recommendations should be used with clinical justification and judgement given the lack of evidence-based research for podiatry management of CIPN.

It would be of benefit to conduct further studies with robust methodology on the effectiveness of various non-pharmacological interventions for management of CIPN. Moreover, encouragement and inclusion of podiatrists in the multidisciplinary care of cancer survivors would be ideal given the key role podiatrists play in the care of people with CIPN-specific lower limb problems.

## Conclusions

This is the first study conducted in the field of podiatry that gathered consensus and agreement amongst experienced podiatrists on the management of people with CIPN. The statements gathered from the Delphi survey were compiled into clinical recommendations that reflect the expert opinion of experienced podiatrists in Australia for the care of people with CIPN. These recommendations, which cover subjective history, objective assessment, and management of people with CIPN, will serve to guide podiatrists in clinical practice within Australia.

## Supplementary Information

**Additional file 1.**

**Additional file 2.**

## Data Availability

All raw anonymous responses are available upon reasonable request to the authors.

## Abbreviations

ADLs: Activities of daily living
CDMP: Chronic disease management plan
CIPN: Chemotherapy Induced Peripheral Neuropathy
CREDES: Conducting and REporting of DElphi Studies
EMG: Electromyography
EORTC CIPN 20: The European Organization for Research and Treatment of Cancer as Chemotherapy-Induced Peripheral Neuropathy 20
EORTC QLQ-C30: The European Organization for Research and Treatment of Cancer the Quality-of-Life Questionnaire
FACT: Functional Assessment of Cancer Therapy
LOPS: Loss Of Protective Sensation
NCI-CTCAE: National Cancer Institute Common Terminology Criteria for Adverse Events
QOL: Quality of Life
TNS: Total Neuropathy Score
VAS: Visual Analogue Score
