# Supplementary material for: Development of the consensus-based recommendations for Podiatry care of Neuropathy In Cancer Survivors (PodNICS): a Delphi consensus study of Australian podiatrists
Source: J Foot Ankle Res. 2023 Jun 9;16:33. doi: 10.1186/s13047-023-00632-0 (PMC10251566; doi:10.1186/s13047-023-00632-0)
Supplement: Supplementary file 1 — Additional file 1. [file 13047_2023_632_MOESM1_ESM.pdf]

Additional file 1- Delphi Round 1 Questionnaire

University of South Australia

Podiatry management for Chemotherapy Induced Peripheral Neuropathy (CIPN)

Introduction to Delphi Round 1

Thank you for participating in the Delphi survey for consensus on the podiatry management for chemotherapy Induced Peripheral Neuropathy (CIPN).

Please note that this is Round 1 of the Delphi survey and is the most comprehensive section where we gather as much information as possible – the subsequent rounds will be briefer.

Please note that you can stop the survey at any time and come back to it later as long as you are on the same computer and same log on session (i.e. you have not closed the window). However, be aware that the page you are working on will be 'blanked' therefore it is best to pause (if you wish to) at the beginning of a new page.

Please contact Sindhrani Dars at sindhrani.dars@mymail.unisa.edu.au (Mobile: 0414 710 226) or Dr Helen Banwell at helen.banwell@unisa.edu.au (Mobile: 0417 822 997) for any queries or concerns.

* 1. Have you already read and understood the participant information sheet sent to you via email and agree to provide consent to participate in this research?

- ◯ Yes
- ◯ No

University of South Australia

Podiatry management for Chemotherapy Induced Peripheral Neuropathy (CIPN)

Unable to continue

You have indicated that you do not consent to proceed. If this was a mistake, please use the back button below, otherwise we ask you to contact us if you would like to discuss your concerns with this project.

Sindhrani Dars at <sindhrani.dars@mymail.unisa.edu.au> or (Mobile: 0414710226)

Podiatry management for Chemotherapy Induced Peripheral Neuropathy (CIPN)

Eligibility to participate

* 2. Please indicate which of the following inclusion criterion/criteria you meet to be eligible for this research (please select all that applies).

- [ ] Someone who has worked within a highrisk foot clinic for 5 years or more, or chronic disease focused private setting for 10 years or more.
- [ ] Someone who holds an academic position teaching podiatry led neuropathy-based management techniques
- [ ] Someone who has published research on conservative intervention techniques for lower limb neuropathy within the last 5 years
- [ ] All of the above
- [ ] None of the above

Podiatry management for Chemotherapy Induced Peripheral Neuropathy (CIPN)

Survey Overview

There are three sections to this survey.

Section 1 is about you. All this information will remain anonymous but lets the research team understand who the experts are, to confirm the eligibility criteria and to report on the traits of the experts (anonymous) in the research publication.

Section 2 is aimed at determining the common presenting symptoms and clinical factors observed by podiatrists for people seeking podiatry services after chemotherapy.

Section 3 asks what management and intervention strategies you use, if any, for symptoms associated with Chemotherapy Induced Peripheral Neuropathy (CIPN) and what common symptoms and signs are seen in the lower limb post-chemotherapy.

Please note that the focus of this study is on neuropathy caused by chemotherapy.

Please begin Section 1 by clicking on the Next button below.

* 3. Please add your name and email address below. This information is only used by the research team to return your responses (if requested) from this round and send Round 2 once we've collated all the information.

Podiatry management for Chemotherapy Induced Peripheral Neuropathy (CIPN)

Section 1 – Participant's characteristics

Please answer the following questions. A reminder that this information will not be shared beyond the research team.

4. Your date of birth (Optional)

Date

Date

DD/MM/YYYY

5. Your gender

○ Male

○ Female

○ Prefer not to say

6. How many years have you been practising as a podiatrist (in full time equivalence)?

7. What was your original qualification in podiatry?

- ◯ Certificate
- ◯ Advanced certificate
- ◯ Bachelors degree
- ◯ Bachelors degree with honours
- ◯ Other (please specify)

8. Where did you obtain your original qualification in podiatry?

- ◯ Charles Sturt University, Australia
- ◯ Curtin University, Australia
- ◯ LaTrobe University, Australia
- ◯ Queensland University of Technology, Australia
- ◯ University of South Australia, Australia
- ◯ University of Western Australia, Australia
- ◯ University of Western Sydney, Australia
- ◯ University of New Castle, Australia
- ◯ James Cook University, Australia
- ◯ Auckland University of Technology, Australia
- ◯ Other (please specify)

9. Have you completed further tertiary study (i.e.: Graduate Diploma, Masters, PhD etc)?

- ◯ No
- ◯ Yes (please specify)

10. What is your highest academic qualification?

- ◯ Graduate certificate
- ◯ Graduate diploma
- ◯ Masters by coursework
- ◯ Masters y research
- ◯ Professional Doctorate
- ◯ Doctorate by research (PhD)
- ◯ Other (please specify)

11. What is your primary position?

- ◯ Clinician
- ◯ Academic
- ◯ Researcher
- ◯ Other (please specify)

12. On average, how many hours a week do you work at your primary position?

13. What is your secondary position?

- ◯ I do not hold a secondary position
- ◯ Clinician
- ◯ Academic
- ◯ Researcher
- ◯ Other (please specify)

14. On average, how many hours a week do you work at your secondary position?

15. Do you hold any other positions?

- ◯ No
- ◯ Yes (please specify)

16. Where do you primarily practice?

- ◯ Australian Capital Territory, Australia
- ◯ New South Wales, Australia
- ◯ Northern Territory, Australia
- ◯ Queensland, Australia
- ◯ South Australia, Australia
- ◯ Tasmania, Australia
- ◯ Victoria, Australia
- ◯ Western Australia, Australia
- ◯ Other (please specify)

17. Please estimate how many patients/clients you would see in a standard working week?

End of Section 1.
Please click on the next button to start Section 2.

Podiatry management for Chemotherapy Induced Peripheral Neuropathy (CIPN)

Section 2 – Clinical factors and presentation of people with CIPN

This section is aimed at determining the common presenting signs and symptoms and the clinical factors observed by podiatrists for people seeking podiatry services after chemotherapy.

18. Have you seen patients/clients with Chemotherapy Induced Peripheral Neuropathy (CIPN) in the last 12 months?

- ◯ Yes
- ◯ No (Skip to Q21)

19. Can you estimate how many patients/clients you have seen with CIPN in the last three months?

- ◯ 0-5
- ◯ 5-10
- ◯ 10-15
- ◯ 15-20
- ◯ 20+

20. For the people you see with CIPN, what is the most likely source of referral? (more than one option can be selected)

- [ ] General Practioner
- [ ] Oncologist
- [ ] Nurse Practioners
- [ ] Self-referred
- [ ] Allied health professionals e.g. Physiotherapist, Exercise Physiologist, Speech pathologist etc.
- [ ] Can you list any further sources of referral for people with CIPN have you received?

21. Please identify the funding source which is most likely used for podiatry services by people attending your practice with chemotherapy induced peripheral neuropathy.

- ○ Publicly funded (e.g. attending a hospital or community based practice)
- ○ Private health
- ○ Chronic disease management plan CDMP/Medicare
- ○ National DIsability Insurance Scheme (NDIS)
- ○ Other (please specify)

22. From your experience or knowledge, what are the common lower limb specific presenting signs and symptoms of people with CIPN. Please list as many as you can, include details where necessary (e.g. if stating 'neurological symptoms', can you please identify which symptoms they most frequently report).

23. From your experience or knowledge, are there any clinical factors or presentations of people with CIPN that appear different to other causes of neuropathy such as diabetes. Or any clinical factors/presentations that you think are unique to neuropathy caused by chemotherapy.

24. In your experience, what percentage of patients/clients with CIPN arrive with an existing diagnosis of CIPN? (e.g. have been assessed by their GP, oncologist, cancer nurse etc.,)

25. In the past 12 months, can you estimate how many, if any, patients/clients you have diagnosed with CIPN that were unaware of their neuropathy.

26. Is there any other information that you would like to add for presentation and clinical factors of people with CIPN receiving podiatry services?

You are nearly there! One more section and you have finished.

Podiatry management for Chemotherapy Induced Peripheral Neuropathy (CIPN)

Section 3 – Podiatry management of Chemotherapy Induced Peripheral Neuropathy (CIPN)

This section is focused on determining how you would manage Chemotherapy Induced Peripheral Neuropathy (CIPN) during the course of your normal practice.

27. In the course of your normal practice, please describe how do you (routinely) assess for neuropathy?

28. In the course of your normal practice, how confident are you that the following assessment can establish the presence of CIPN;

	Very Confident	Confident	Neutral	Unlikely	Very Unlikely
10gm Monofilament test	◯	◯	◯	◯	◯
Tuning fork assessment	◯	◯	◯	◯	◯
Two-point discrimination test	◯	◯	◯	◯	◯
Biothesiometer or Neurothesiometer	◯	◯	◯	◯	◯
Self-reported neurological symptoms	◯	◯	◯	◯	◯
Nerve conduction study	◯	◯	◯	◯	◯
GP notification	◯	◯	◯	◯	◯
Patient reported diagnosis	◯	◯	◯	◯	◯
Oncologist notification	◯	◯	◯	◯	◯
Presence of wounds/ulcers due to unfelt trauma	◯	◯	◯	◯	◯
Presence of comorbidities likely to worsen neuropathy e.g. diabetes	◯	◯	◯	◯	◯

Other (please specify)

29. In course of your normal practice, describe what management options and interventions for CIPN you are most likely to use in the **first instance**? Please provide as much information as you can.

30. In course of your normal practice, describe what **other** management options and interventions for CIPN do you use?
Again, please provide as much information as possible (e.g. if you use two or more strategies together, please indicate this).

31. On average, how often (in weeks) do you review your patients with CIPN.

32. Given there is little evidence to date on the podiatry management of CIPN, please indicate why you use your choice of management options listed above (e.g. success from experience, learnt skills from senior/experienced podiatrists, learnt from other professionals as oncologists, anecdotal etc).

Thank you for taking time to complete this Round 1 of the Delphi survey. Your time and participation are really appreciated.

University of South Australia

Podiatry management for Chemotherapy Induced Peripheral Neuropathy (CIPN)

Thank you

Thank you again and we will be in touch for Round 2 around 4 weeks after the closing date of this round **(23rd May 2022, 5pm, COB).**

Mrs Sindhrani Dars - Chief Investigator

Supervisors – Dr Helen Banwell, Dr Liz Buckley, Dr Kerrilyn Beckmann and Professor David Roder.

If you have any queries, please contact Sindhrani Dars at sindhrani.dars@mymail.unisa.edu.au, 0414710226.
