# Supplementary material for: Development of the consensus-based recommendations for Podiatry care of Neuropathy In Cancer Survivors (PodNICS): a Delphi consensus study of Australian podiatrists
Source: J Foot Ankle Res. 2023 Jun 9;16:33. doi: 10.1186/s13047-023-00632-0 (PMC10251566; doi:10.1186/s13047-023-00632-0)
Supplement: Supplementary file 2 — Additional file 2. [file 13047_2023_632_MOESM2_ESM.pdf]

Additional file 2 – Statements sent for review in subsequent rounds and the rejected statements.

Statements sent for review in subsequent rounds.

Table 1 - Themed statements from comments to open ended questions and 50-69% agreement on Likert scale in Round 1 and 2 sent for review in Round 2 and 3 respectively

Category	Statement	Round of Delphi	(n = X) no. of participants contributing to the themed statement, % for Likert statements
**Clinical factors and presentation of people with CIPN**			
**Common presenting signs and symptoms of people with CIPN**	Loss of protective sensation (LOPS) and Loss of proprioception	One	(n = 6/19)
	Muscle weakness, motor control difficulties and balance disturbances leading to gait changes (e.g., shuffling, reduced velocity etc.,) and reduced physical activity	One	(n = 5/19)
	Nail changes including but not limited to: onychogryphosis, onychomycosis, Onychauxis, Onychocryptosis and nails that are friable, dystrophic, have reduced growth and flaking	One	(n = 5/19)
	Autonomic changes including but not limited to: blood pressure and temperature regulation (cold feet/Raynaud's phenomenon)	One	(n = 4/19)
	Skin changes including but not limited to: atrophy + rubor, skin shedding/peeling, dry skin, moccasin type cracking and painful blistering	One	(n = 3/19)
	Presence of neuropathic foot ulceration with or without infection	One	(n = 3/19)
	Motor control difficulties of upper limbs leading to a reduction in lower limb self-care (e.g., ability to cut own nails)	One	(n = 1/19)

	Muscle weakness, motor control difficulties and balance disturbances leading to gait changes (e.g., shuffling, reduced velocity etc.,) and reduced physical activity	Two	(n=10/17) – 58.8% agreement
**Clinical factors or presentation unique to CIPN**	Sudden (acute) onset and quick progression of symptoms	One	(n = 6/19)
	In some people, symptoms may improve or resolve with chemotherapy dose reduction or cessation	One	(n = 5/19)
	Painful presentations appear more prevalent in CIPN than other forms of neuropathy	One	(n = 4/19)
	Skin anhidrosis with rubor, skin shedding and increased injuries	One	(n = 3/19)
	Similar symptoms may be present in hands and feet that may quickly spread proximally to upper arms and legs	One	(n = 3/19)
	Widely distributed symptoms, often affecting other body functions (e.g., speech and bladder function)	One	(n = 3/19)
	Loss of proprioception/affected balance more prevalent than in other types of neuropathies	One	(n = 2/19)
	Nail deformities and Infected in-growing toenails common	One	(n = 2/19)
	Can occur in conjunction with hand and foot syndrome	One	(n = 1/19)
	Patients are generally aware of the symptoms due to chemotherapy education	One	(n = 1/19)
	Hard to differentiate from diabetes related neuropathy	One	(n = 1/19)
	Skin anhidrosis with rubor, skin shedding and increased injuries	Two	(n=11/17) – 64.7% agreement
	Infected in-growing toenails are more common	Two	(n=10/17) – 58.8% agreement
	Nail deformities are more common	Two	(n=9/17) – 53% agreement
**Additional information on Clinical factors and presentation of people with CIPN.**	Numbness without loss of protective sensation (LOPS)	Two	(n=1/17)
	Presents in a glove and stocking distribution	Two	(n=1/17)
	Can reduce patient's confidence and engagement in physical activity	Two	(n=1/17)
	Presents with reduced dorsiflexion & heel strike, increasing forefoot pressure	Two	(n=1/17)
	Presents with drop foot, neuropathic gait or 'high steppage' gait	Two	(n=1/17)
	More likely to impact on mental health or wellbeing (than other forms of peripheral neuropathy)	Two	(n=1/17)
	Clawed or retracted digits	Two	(n=1/17)
**Diagnosis and Assessment of CIPN**			
	Deep tendon reflexes	One	(n = 5/18)

**Diagnostic and Assessment tools routinely utilised**	Muscle strength and Joint Range of Motion	One	(n = 5/18)
	Dynamic balance/proprioception testing such as Single Leg Stance (SLS)/sway test	One	(n = 5/18)
	Cotton bud for light pressure	One	(n = 3/18)
	Thermal (hot/cold) sensation	One	(n = 3/18)
	Gait and plantar pressure investigations	One	(n = 3/18)
	Biothesiometer/Neurothesiometer	One	(n = 2/18)
	Neurotip (40g) or pin prick (sharp/blunt)	One	(n = 2/18)
	Two-point discrimination	One	(n = 1/18)
	The Ipswich touch test	One	(n = 1/18)
	Diabetes foot assessment	One	(n = 1/18)
	Footwear assessment	One	(n = 1/18)
	Changes to skin integrity following chemotherapy	One	(n = 1/18)
	Inspect for callus, pre-ulcerative lesions and ulcers	One	(n = 1/18)
	Dynamic balance/proprioception testing such as Single Leg Stance (SLS)/sway test	Two	(n=11/17) – 64.7% agreement
	Deep tendon reflexes	Two	(n=11/17) – 64.7% agreement
	Footwear assessment	Two	(n=11/17) – 64.7% agreement
	Diabetes foot assessment	Two	(n=10/17) – 58.8% agreement
**Assessment tools/pathways that could confirm CIPN diagnosis**	GP notification	One	(n=12/18) – 66.6% agreement
	Patient reported diagnosis	One	(n=11/18) – 61.1% agreement
	Nerve conduction study	One	(n=11/18) – 61.1% agreement
	Biothesiometer or Neurothesiometer	One	(n=11/18) – 61.1% agreement
	Patient reported signs and symptoms/outcomes using validated questionnaires e.g., Visual Analogue Scale (VAS)	One	(n=1/18)
	Presence of wounds in absence of Vascular compromise	One	(n = 1/18)
	Pathology tests to rule out other causes of neuropathy as Diabetes, Alcoholism, nutritional deficiency etc.	Two	(n=2/17)
	Electromyography (EMG) by Neurologist	Two	(n=1/17)
	**Podiatry Management of CIPN**		
**Podiatry Management of CIPN**	Footwear assessment and education (properly fitting, supportive, lightweight and comfortable)	One	(n=12/18)
	Management and offloading of pressure lesions, wounds or blisters	One	(n=11/18)

	Assessing that pharmacological pain management is in place and educate on non-pharmacological pain management modalities (heat packs, wheat bags, topical capsaicin etc)	One	(n=10/18)
	Regular footcare (nails including ingrowing toenails and skin including hyperkeratosis)	One	(n=7/18)
	Engagement with possible referral to other allied health professionals as required (e.g Physiotherapist, Occupational therapist, Exercise physiologist, psychologist and pain management clinics)	One	(n=4/18)
	A targeted personalised management plan appropriate for severity of the condition and considering patient's finances	One	(n=4/18)
	Advising on appropriate physical activity or exercise regimes	One	(n=4/18)
	Taping painful intact skin areas like toes (kinesio/rocktape/opsite) to help with neuropathic pain	One	(n = 3/18)
	Communication with GP and oncology team, particularly where foot-related symptoms are severe	One	(n=3/18)
	Advise on escalation of care if needed in case of development of foot infection or ulceration	One	(n=1/18)
	Advice on lifestyle changes including alcohol, smoking, and diet	One	(n=1/18)
	Discuss options for use of mechanical aids like walkers and braces	One	(n=1/18)
	Discussion regarding their driving ability	One	(n=1/18)
	Alternative or adjuvant treatment e.g., acupuncture	One	(n = 1/18)
	Discuss options for use of mechanical aids like walkers and braces	Two	(n=11/16) – 68.7% agreement
	Advising on appropriate physical activity or exercise regimes	Two	(n=9/16) – 56% agreement
	Advise on lifestyle changes including alcohol, smoking, and diet	Two	(n=8/16) – 50% agreement
	Discussion regarding their driving ability	Two	(n=8/16) – 50% agreement
**Additional information on Podiatry**	Referral to support groups including appropriate online resources such as guides for people living with or surviving cancer	Two	(n=2/16)
	Help access financial support/economical treatments e.g. via Chronic disease management plan	Two	(n=1/16)

**management of CIPN**	Podiatrists can advise or complete alternate or adjunct treatments including (but not limited to) Cryotherapy, scrambler therapy, acupuncture, massage therapy etc	Two	(n=1/16)
	Referral to mental health professionals and/or helping access resources for it e.g. Finding my way (online self-help for people with cancer)	Two	(n=1/16)
	Podiatrist-based resources on the management of CIPN are required	Two	(n=1/16)
	Multidisciplinary care is essential	Two	(n=1/16)

Statements rejected in Round 2 and 3

Table 2 - Rejected statements for not meeting the required minimum agreement of 50% (Round 1 and 2) or 70% (Round 3)

Category	Statement	Round rejected	(n = X) - % consensus/agreement
**Clinical factors and presentation of people with CIPN**			
**Frequently reported signs and symptoms of CIPN**	Presence of neuropathic foot ulceration with or without infection	Two	(n=6/17) – 35% agreement
	Motor control difficulties of upper limbs leading to a reduction in lower limb self-care (e.g. ability to cut own nails)	Two	(n=6/17) – 35% agreement
	Muscle weakness, motor control difficulties and balance disturbances leading to gait changes (e.g. shuffling, reduced velocity etc.,) and reduced physical activity	Three	(n=9/16) – 56% agreement
**Clinical factors or presentation unique to CIPN**	Painful presentations appear more prevalent in CIPN than other forms of neuropathy	Two	(n=4/17) – 23.5% agreement
	Similar symptoms may be present in hands and feet that may quickly spread proximaly to upper arms and legs	Two	(n=7/17) – 41% agreement
	Loss of proprioception/affected balance more prevalent than in other types of neuropathy	Two	(n=3/17) – 18% agreement
	Widely distributed symptoms, often affecting other body functions (e.g. speech and bladder function)	Two	(n=6/17) – 35% agreement

Category	Statement	Round rejected	(n = X) - % consensus/agreement
	Can occur in conjunction with hand and foot syndrome	Two	(n=2/17) – 12% agreement
	Patients are generally aware of the symptoms due to chemotherapy education	Two	(n=7/17) – 41% agreement
	Hard to differentiate from diabetes related neuropathy	Two	(n=4/17) – 23.5% agreement
	Infected in-growing toenails are more common	Three	(n=9/16) – 56% agreement
	Nail deformities are more common	Three	(n=10/16) – 62.5% agreement
**Additional information on Clinical factors and presentation of people with CIPN**	Numbness without loss of protective sensation (LOPS)	Three	(n=10/16) – 62.5% agreement
	Presents in a glove and stocking distribution	Three	(n=10/16) – 62.5% agreement
	Presents with reduced dorsiflexsion & heel strike, increasing forefoot pressure	Three	(n=2/16) – 12.5% agreement
	Presents with drop foot, neuropathic gait or 'high steppage' gait	Three	(n=3/16) – 19% agreement
	More likely to impact on mental health or wellbeing (than other forms of peripheral neuropathy)	Three	(n=6/16) – 37.5% agreement
	Clawed or retracted digits	Three	(n=6/16) – 37.5% agreement
**Diagnosis and Assessment of CIPN**			
**Diagnostic and Assessment tools routinely utilised**	Cotton bud for light pressure	Two	(n=6/17) – 35% agreement
	Thermal (hot/cold) sensation	Two	(n=8/17) – 47% agreement
	Gait and plantar pressure investigations	Two	(n=7/17) – 41% agreement
	Biothesiometre/neurothesiometre	Two	(n=7/17) – 41% agreement
	Neurotip (40g) or pin prick (sharp/blunt)	Two	(n=7/17) – 41% agreement
	Two point discrimination	Two	(n=7/17) – 41% agreement
	The Ipswich touch test	Two	(n=6/17) – 35% agreement
	Dynamic balance/proprioception testing such as Single Leg Stance (SLS)/sway test	Three	(n=11/16) – 69% agreement
**Assessment tools/pathways that could confirm diagnosis**	Two-point discrimination test	Two	(n=7/17) – 41% agreement
	Presence of wounds in absence of Vascular compromise	Two	(n=8/17) – 47% agreement
**Additional information on the diagnosis and assessment of CIPN**	Pathology tests to rule out other causes of neuropathy as Diabetes, Alcoholism, nutritional deficiency etc.	Three	(n=10/16) – 62.5% agreement
	Electromyography (EMG) by Neurologist	Three	(n=4/16) – 25% agreement

Category	Statement	Round rejected	(n = X) - % consensus/agreement
**Podiatry Management of CIPN**			
**Podiatry Management of CIPN**	Taping painful intact skin areas like toes (kinesio/rocktape/opsite) to help with neuropathic pain	Two	(n=6/16) – 37.5% agreement
	Alternative or adjuvant treatment e.g. acupuncture	Two	(n=5/16) – 31% agreement
**Additional information on Podiatry management of CIPN**	Referral to support groups including appropriate online resources such as guides for people living with or surviving cancer	Three	(n=9/16) – 56% agreement
	Help access financial support/economical treatments e.g. Via Chronic disease management plan	Three	(n=3/16) – 19% agreement
	Podiatrists can advise or complete alternate or adjunct treatments including (but not limited to) Cryotherapy, scrambler therapy, acupuncture, massage therapy etc.	Three	(n=9/16) – 56% agreement
	Referral to mental health professionals and/or helping access resources for it e.g. Finding my way (online self-help for people with cancer)	Three	(n=9/16) – 56% agreement
